# Designing eHealth Applications to Reduce Cognitive Effort for Persons With Severe Mental Illness: Page Complexity, Navigation Simplicity, and Comprehensibility

**DOI:** 10.2196/humanfactors.6221

**Published:** 2017-01-05

**Authors:** Armando J Rotondi, Michael R Spring, Barbara H Hanusa, Shaun M Eack, Gretchen L Haas

**Affiliations:** ^1^ Mental Illness Research, Education and Clinical Center (MIRECC) VA Pittsburgh Health Care System Department of Veterans Affairs Pittsburgh, PA United States; ^2^ School of Medicine Center for Behavioral Health and Smart Technology University of Pittsburgh Pittsburgh, PA United States; ^3^ School of Medicine Clinical and Translational Sciences Institute University of Pittsburgh Pittsburgh United States; ^4^ School of Medicine Department of Critical Care Medicine University of Pittsburgh Pittsburgh United States; ^5^ Information Science and Technology School of Information Sciences University of Pittsburgh Pittsburgh, PA United States; ^6^ School of Social Work University of Pittsburgh Pittsburgh, PA United States; ^7^ School of Medicine Department of Psychiatry University of Pittsburgh Pittsburgh, PA United States

**Keywords:** Internet technology, mobile application, cognitive impairment, eHealth, eHealth design, e-mental health, schizophrenia, severe mental illness, usability, website design

## Abstract

**Background:**

eHealth technologies offer great potential for improving the use and effectiveness of treatments for those with severe mental illness (SMI), including schizophrenia and schizoaffective disorder. This potential can be muted by poor design. There is limited research on designing eHealth technologies for those with SMI, others with cognitive impairments, and those who are not technology savvy. We previously tested a design model, the Flat Explicit Design Model (FEDM), to create eHealth interventions for individuals with SMI. Subsequently, we developed the design concept *page complexity*, defined via the design variables we created of distinct *topic areas*, distinct *navigation areas*, and number of columns used to organize contents and the variables of text reading level, text reading ease (a newly added variable to the FEDM), and the number of hyperlinks and number of words on a page.

**Objective:**

The objective of our study was to report the influence that the 19 variables of the FEDM have on the ability of individuals with SMI to use a website, ratings of a website’s ease of use, and performance on a novel usability task we created termed as *content disclosure* (a measure of the influence of a homepage’s design on the understanding user’s gain of a website). Finally, we assessed the performance of 3 groups or dimensions we developed that organize the 19 variables of the FEDM, termed as page complexity, navigational simplicity, and comprehensibility.

**Methods:**

We measured 4 website usability outcomes: ability to find information, time to find information, ease of use, and a user’s ability to accurately judge a website’s contents. A total of 38 persons with SMI (chart diagnosis of schizophrenia or schizoaffective disorder) and 5 mental health websites were used to evaluate the importance of the new design concepts, as well as the other variables in the FEDM.

**Results:**

We found that 11 of the FEDM’s 19 variables were significantly associated with all 4 usability outcomes. Most other variables were significantly related to 2 or 3 of these usability outcomes. With the 5 tested websites, 7 of the 19 variables of the FEDM overlapped with other variables, resulting in 12 distinct variable groups. The 3 design dimensions had acceptable coefficient alphas. Both navigational simplicity and comprehensibility were significantly related to correctly identifying whether information was available on a website. Page complexity and navigational simplicity were significantly associated with the ability and time to find information and ease-of-use ratings.

**Conclusions:**

The 19 variables and 3 dimensions (page complexity, navigational simplicity, and comprehensibility) of the FEDM offer evidence-based design guidance intended to reduce the cognitive effort required to effectively use eHealth applications, particularly for persons with SMI, and potentially others, including those with cognitive impairments and limited skills or experience with technology. The new variables we examined (topic areas, navigational areas, columns) offer additional and very simple ways to improve simplicity.

## Introduction

The closing of long-term hospitals and the increasingly abbreviated stays in acute care facilities leave persons with severe mental illness (SMI) more dependent on outpatient services and self-care to support their community well-being. Prevailing in-person mental health service delivery models have resulted in a situation where many treatments, proven efficacious over the past 30 years, are not readily available [1-4]; available services rarely meet the established standards for care [5-8]; only 40%-50% receive specialty mental health care in a given year [6-8]; and only 4%-15% receive even minimally adequate mental health treatment—far short of evidence-based standards [7,9,10]. Consequently, the majority of persons with SMI live in communities where they and their families receive few services, and those services fall substantially short of the established standards for care [11,12]. Clearly there is a need for cost-effective ways to increase receipt of care and successfully disseminate evidence-based interventions to communities [11].

eHealth technologies are being used increasingly in general medical care [13-16] and to a lesser, though growing, extent in mental health treatment [17-20] as a way to improve service receipt and reduce illness burden. In this study, eHealth refers to the use of consumer-facing information and communication technologies to support health [21,22], with a focus on Web-based and mobile phone apps. As eHealth technologies become more prominent, the dearth of models to effectively design them for persons with SMI [21,22], and others with cognitive impairments, will result in significant obstacles to obtaining services for these individuals and exacerbate already inadequate receipt of services [6]. Several investigators have identified the difficulty that those with SMI have in using websites designed for the general public [22-24]. In response, research has been conducted to develop designs appropriate for those with SMI [25-28].

As part of our prior work, we developed an empirically supported design model to create eHealth technologies that persons with SMI, with little or no prior technology experience, could use effectively [23]. The resulting nascent model, termed the Flat Explicit Design Model (FEDM), proved to be quite effective. Using this model, we created a Web-based intervention, termed Schizophrenia Online Access to Resources (SOAR), to provide in-home multifamily psychoeducational treatment to persons with schizophrenia and their family members [23]. The intervention website was highly valued, frequently used, and had significant effects on important outcomes (eg, reducing positive symptoms) [29,30]. Its design proved to be quite effective. In tests comparing this website to public websites for persons with SMI, those with SMI took less time to find contents, had greater success finding contents, and rated it easier and less frustrating to use [24].

This study evaluated several additions to the design model (FEDM, Textbox 1) used to create SOAR. Observations during previous usability studies suggested that some individuals with SMI had difficulty scanning a page effectively for content and creating a mental model of the layout and organization of a screen’s contents [23,28]. Generally, these are important requirements to effectively navigate standard eHealth apps. Others have found that individuals with schizophrenia are not able to use websites that are well designed, but intended for the general public [22]. These observations, coupled with previous findings, and knowledge of the cognitive deficits associated with SMI led us to develop the novel concept of *page complexity* as potentially important to the cognitive effort required to comprehend and effectively use an eHealth app. In elaborating a model of page complexity, we developed the new design constructs of distinct *topic areas* and *navigation areas*. Additionally, our experience led us to speculate that the number of columns used to organize contents, number of words and hyperlinks on a page, and the text’s reading level and simplicity (ie, reading ease) would also influence the cognitive effort required for comprehension and navigation and thus, an app’s usability. We hypothesized that these constructs and variables would have an effect on the ability of persons with SMI to navigate eHealth technologies. This study was designed to test: these new constructs; the initial validity of our concept of page complexity; a set of variables to define page complexity; the addition of a new variable, reading ease, to the FEDM; and three functional dimensions we developed to organize the 19 variables of the FEDM. These dimensions were designed to have practical implications for creating effective designs. The study had persons with schizophrenia perform tasks on 5 public mental health websites during which data were collected on the ability to find contents, time to find contents, subjective evaluation of the websites, and influence of the homepage designs on a user’s understanding of a website.

**Dimensions and variables of the Flat Explicit Design Model (FEDM): page complexity, navigation simplicity, and comprehensibility.**

Dimension 1: page complexity

- Number of navigation areas: A minimal number of navigation areas per screen preferably in 1 location and prominent on the screen.
- Number of topic areas: A minimal number of independent topic areas on a screen.
- Number of columns: A minimal numbers of columns on a screen that present page contents.
- Depth of hierarchy: A minimal number of screens or pages that need to be navigated in order to find desired contents.
- Number of themes: A limited number of disparate themes or topics on any 1 screen.
- Display distractions: A plain presentation that minimizes distracting and superfluous content (eg, decorative displays or images).
- Reading ease: Text should use words that are understandable and simple sentence structures.
- Number of hyperlinks: Use of hyperlinks in navigation areas in order to decrease the number of navigation areas on a page. In the use of these, the need for minimal depth of pages also must be considered.
- Use of low-level hyperlink categories to navigate to contents: The organization of the hyperlinks to an app’s contents should be accomplished by using low-level categories, that is, less abstract categories, closer conceptually, as well as closer in the navigational hierarchy, to final destination contents, and thus there should be relatively more hyperlink categories on navigation screens, particularly the home screen.

Dimension 2: navigation simplicity

- Toolbar: A single constant navigational tool bar is used to improve comprehension and navigation.
- Explicit hyperlink labels: Navigational elements should not need inference or interpretation to understand. Hyperlinks, icons, headings, and labels should be explicit.
- Hyperlink location: Navigational elements should be placed in a minimum of different locations and preferably in the upper left of a page, where users begin searching.
- Introductory content location: Minimize introductory text before the hyperlinks that lead to an app’s contents.

Dimension 3: comprehensibility

- Number of words: Minimal words should be used, but they must convey concepts without the need to interpret meaning.
- Page Length: Emphasis should be placed on scrolling down a page for additional content versus navigating to another page. A longer page is more complex than a shorter page, but that is better than making the page shorter by having users navigate to another page.
- Memory aids: Memory aids should be used, for example, pop-up menus, to facilitate navigation.
- Reading Level: A low reading level should be used.
- Inference: There should be minimal need to infer meaning or think abstractly in order to understand the written content presented.
- Use of Dialect: Words used should employ target groups’ vernacular and vocabulary.

## Methods

### Participant Recruitment and Selection Criteria

Participants were recruited from 6 community psychiatric rehabilitation outpatient centers, which provided day treatment programs. To receive services, one had to have an SMI diagnosis and appropriate insurance (eg, Medicare). Staff identified individuals with proper diagnoses and discussed the study with them. Those who were interested were told when study staff would be there (ie, to conduct usability testing). Enrollment criteria were as follows: age ≥18 years; a chart diagnosis of schizophrenia or schizoaffective disorder; physical ability to read the screen of a computer and use a mouse; and ability to read at a 5th grade level. There were no requirements for prior computer, Internet, or website use. The research protocol was approved by the University of Pittsburgh’s institutional review board.

### Participant Background Information

The Global Assessment Scale (GAS) [31] was used by study staff who conducted the usability testing to rate each participant’s level of functioning. All staff were formally trained and experienced at rating persons with schizophrenia using the GAS. Sociodemographic and computer experience information were also collected (Table 1) —one participant did not answer the computer access question.

### Choice of Websites for Evaluation

Five websites that included information on schizophrenia were chosen for the study. The website SOAR [30] was developed to deliver family psychoeducational treatment using the FEDM. Its address was not provided because it is currently under study. The other 4 were identified using Google to search for the word schizophrenia and by searching the Google directory. The websites were chosen to represent variation on important design variables:Schizophrenia.com (started in 1995), one of the first websites listed in the search results, provides support groups, science-based information, and discussion forums for individuals and families; the website of the National Alliance on Mental Illness (started in 1979), designed to provide education, advocacy, help, and public awareness to individuals and family members; Chovil.com (started in 1997), was created by an individual with schizophrenia and, thus, may contain design insights not commonly found in other sites; and the National Mental Health Association-Mental Health America (founded in 1909) has a website that focuses on individuals with mental illness and provides resources to promote mental health wellness. All websites were downloaded and cached locally to ensure that their contents remained static throughout the study.

### Training of Testers

All testers were mental health professionals and had experience working with individuals with SMI. They also all had experience in providing training to individuals with schizophrenia on how to use computers and navigate websites from previous research projects. Two of the authors (AR and SE) provided training to testers and supervised them as they conducted the testing procedures on each other and other staff. Additional supervision was provided during the data collection for this study.

### Participant Training and Testing

To ensure the basic knowledge needed to navigate the websites, each participant was taken through a brief tutorial using 3 preselected public websites (different from the 5 tested) to teach the basic elements that would be needed to navigate the 5 websites being tested. The topics covered included using a mouse, hyperlink text, navigation buttons, pop-up menus, and scroll bar to scroll up and down a page. The longest training took 22 minutes. This was based on tutorials we had used previously [29,32]. All who met eligibility criteria were able to master the use of these basic elements. Training was provided before the testing occurred by the research staff conducting the usability testing.

### Characterizing Each Website’s Homepage Complexity

Each website’s homepage was characterized in terms of 19 variables (Table 2). Reading level (in terms of school year), reading ease (for which higher values represent easier reading ease), and total number of words were determined using the grammar (which utilized the Flesch and Kincaid formulas) [33] and word count functions in Microsoft Word. The number of hyperlinks and columns of contents on the homepage were counted manually. If a homepage had sections with different numbers of columns, a weighted average was calculated by summing the number of columns in each section multiplied by a section’s proportion to the total length of the page. A *topic area* was defined as being a distinct area of a screen or page that was devoted to a given topic or purpose, and it could contain both nonhyperlink and hyperlink text. A *navigation area* was a distinct area of a screen or page that contained 1 or more hyperlinks grouped together by proximity and typically related to one another via a common topic. Many topic areas were composed of text (ie, nonhyperlink text), which introduced the topic of the area, and included 1 or more hyperlinks associated with the topic. If a set of hyperlinks occupied a separate area of the page, was not associated with any text, and represented a distinct topic from other areas in proximity, it was classified as a topic area, and it was also classified as a navigation area.

### Website Evaluation Procedures

The usability testing occurred in private rooms at the psychiatric rehabilitation outpatient centers. Testing was done on our computers. During the testing, to control for learning effects, the order of testing of the 5 websites was varied across participants using a 5×5 Latin square design. Likewise, the order of presentation of tasks within each of the 3 different types of tasks participants performed were varied across individual websites.

Website usability testing was conducted using software, Ergobrowser, (Ergosoft) for recording and timing website navigation. Participants were timed by pressing a special keyboard button, which started and stopped the timing for each subtask. It also recorded which subtask participants were performing.

### Tasks to Evaluate Website Usability Performance

#### Task 1: Homepage Content Disclosure Performance

To assess the effectiveness with which a homepage’s design conveys information about a website’s contents (what we term homepage *content disclosure*), participants were asked to study a homepage for 60 seconds. They could scroll down the page but not use the mouse to open pull-down or pop-up menus. Then, while still being able to look at the page, they decided (using the options of yes or no) whether they thought a website contained information on 7 topics: how schizophrenia is treated, how to find a good psychiatrist, the side effects of medications, the causes of schizophrenia, how to find volunteer work, how to know whether a person has schizophrenia, and how many people have schizophrenia. The 7 topics were chosen so that information was present on 5 of the 7 topics on each website. For analyses the participants were put into one of 2 groups based on the number they got correct: one group was those who got ≤5 correct, the other group was those who got >5 correct. One subject did not complete this task.

#### Task 2: Find Specific Information—Ability and Time

To assess the ability to accurately navigate each website, participants were asked to find content on 3 topics: (1) treatments for schizophrenia, (2) side effects of medications used to treat schizophrenia, and (3) the causes of schizophrenia. Participants were given a maximum of 5 minutes to find the information on each topic. Once a search was over, participants were returned to the homepage to begin the next search. For analysis, we used 2 measures: whether for all 5 tasks the subjects found the correct information and, for the tasks solved correctly, the mean number of seconds needed to correctly find the information. This time was missing for 5 participants.

#### Task 3: Reactions to a Website

Following testing on a website, participants were asked to rate their impressions of the ease of use of a website using a 5-point scale ranging from 1 (“Not at all”) to 5 (“Extremely”).

### Data Analyses

The values obtained for the number of a website’s navigation and topic areas, links, columns and words on the home page, and home page length are included in Table 2. Each variable was dichotomized; that is, the values of each variable were coded into 2 levels: “less complex” versus “more complex” (Table 3). For the 5 websites used in this study, several of the 19 variables overlapped in their variation with other variables, yielding 12 distinct variable groups (Table 3).

We organized the 19 variables of the FEDM into 3 dimensions or *factors*. To make an initial assessment of the validity of these 3 dimensions, coefficient alphas were computed to assess the internal consistency of the variables within the 3 dimensions. A summary score was created for each of the 3 dimensions by adding the dichotomized values of the variables within each dimension. Analyses of the relationships of the usability outcomes to the 19 variables, as well as to the 3 dimensions’ summary scores, were performed using mixed model regressions that accounted for the repeated measures within subjects and across websites. Logistic regressions were used for ability to find all 3 pieces of information versus not finding all 3, and the content disclosure task, which consisted of the number of times each participant correctly identified whether 7 topics were addressed by a website, after only examining the homepage. Performance for the content disclosure task was grouped into ≤5 or >5. Linear regressions were used for mean time to find information, and ease of use. Initially univariate regressions were done followed by multivariable models that included all 12 nonoverlapping FEDM variable groups. Analyses of the summary dimension scores were also completed with mixed effect regressions.

Demographic information, reports of computer experience, and overall function measured by the GAS were summarized with means for continuous variables and frequencies for categorical variables.

## Results

### Principal Findings

The mean age of participants was 47.2 (SD=6.62); 50% (19/38) were females, and 40% (15/38) were African American/Black (Table 1). Twenty participants (53%) reported prior computer use, of which 27% (10/38) had home access, 27% had only nonhome access, and 50% (19/38) had used websites previously.

### Characteristics of the Websites’ Design Complexity

Table 2 presents each of the 5 websites characterized according to the 19 variables of the FEDM. In terms of dimension 1, page complexity, Chovil and SOAR have the lowest design complexity, followed by Schizophrenia.com, with the National Alliance on Mental Illness (NAMI) and the National Mental Health Association (NMHA) having the most complex designs. For example, Chovil has the fewest columns, highest reading ease, and fewest hyperlinks. SOAR is designed using the fewest navigation areas, and both have the fewest topic areas for presenting contents. For dimension 2, navigation simplicity, SOAR and SZ.com have the simplest designs. The other 3 websites are relatively complex on this dimension. With dimension 3, comprehensibility, all websites have some good design characteristics. SOAR has the most, and is the only one to employ the target audience’s dialect and require relatively less inference to understand contents. Chovil has the lowest reading level, followed by Schizophrenia.com, NMHA, and then SOAR, with NAMI having the highest.

### Ability and Time to Find Information, Subjective Ease of Use Ratings, and Content Disclosure Task

Univariate analyses showed that the following 11 variables were predictors of the 4 usability outcomes (content disclosure, ability and time to find contents, ease of website use): few navigation areas,topic areas, columns, shallow depth of hierarchy, few themes, few display distractions, easy to moderate reading ease, constant navigational toolbar, moderate reading level, and explicit hyperlink labels (Table 3). In addition to the aforementioned 11 variables, the ability of a homepage to convey information about a website’s contents to users (ie, content disclosure) was also associated with fewer hyperlinks. The ability to find the 3 pieces of information and the time to find the information were also significantly associated with the following variables: the use of low-level hyperlink categories, having hyperlinks in the upper left of the page, having fewer words on a homepage, shorter homepage length, use of dialect, and minimal inference required. In addition to the aforementioned 11 variables, ease of use ratings were also associated with fewer hyperlinks, use of low-level hyperlink categories, having hyperlinks in the upper left of the page, having the introductory text located after the hyperlinks to contents, having fewer words on a homepage, shorter homepage length, use of dialect,and minimal inference required.

The multivariable analyses indicated that the ability to navigate a website and find the information were significantly related to shallow depth, few themes presented, few display distractions, easy to moderate reading ease (odds ratio, OR=24.5, *P*<.001) and fewer hyperlinks (OR=–0.08, *P*<.001). For the mean time to find the information, shallow depth, moderate reading level (68 vs 47 seconds, *P*<.001) and fewer hyperlinks (68 vs 81 seconds, *P*<.01) remained significant. Users’ ease of website use ratings were significantly related to having fewer navigational areas, topic areas, and columns (3.3 vs 2.9, *P*=.06), shallow depth, few themes presented, few display distractions, easy to moderate reading ease (3.6 vs 2.9, *P*<.001) and fewer hyperlinks (2.8 vs 1.6, *P*<.001). For the content disclosure task, the ability of users to correctly identify the contents of a website after only viewing the homepage was significantly and positively influenced by shallow depth, moderate reading level (OR=3.9, *P*=.001) and having fewer hyperlinks (OR=–3.4, *P*<.001).

**Table 1:** Participant characteristics (N=38).

Variable		n (%)
**Sex**		
	Female	19 (50)
**Age (years)**		
	31-40	6 (15.8)
	41-50	23 (60.5)
	51-59	9 (23.7)
**Race**		
	White	22 (57.9)
	African American, Black	15 (39.5)
	Asian	1 (2.6)
**Education**		
	<High school	6 (15.8)
	High school	12 (31.6)
	Some college or vocational school	14 (36.8)
	College graduate	6 (15.8)
**Overall level of functioning (Global Assessment Scale)**		
	<40	3 (7.9)
	41-61	17 (44.7)
	62-72	17 (44.7)
	73-81	1 (2.6)
**Computer access**		
	At home	10 (26.3)
	Other than home	10 (29.0)
	No access	17 (44.7)
**Hours of computer use/week**		
	None	17 (44.7)
	1-5	13 (34.2)
	>5	8 (21.0)
**Previously accessed websites**		
	Yes	19 (50.0)

**Table 2:** Measures of the Flat Explicit Design Model (FEDM) variables across 5 tested websites.^a^

The 19 design variables of the FEDM			Values for each website on the 19 design variables of the FEDM				
		SOAR^b^	SZ.com^c^	Chovil^d^	NAMI^e^	NMHA^f^
**Dimension 1: page complexity**							
	Number of navigation areas	3	16^g^	4	20^g^	28^g^
	Number of topic areas	4	17^g^	4	17^g^	29^g^
	Number of columns	1.77	2.38^g^	1.58	3.65^g^	2.88^g^
	Shallow depth of hierarchy	Yes	Yes	Yes	No^g^	No^g^
	Few themes presented	Yes	Yes	Yes	No^g^	No^g^
	Few display distractions	Yes	Yes	Yes	No^g^	No^g^
	Reading ease^h^	43.8	43.3	52.3	37.7^g^	39.3^g^
	Number of hyperlinks	97^g^	120^g^	45	132^g^	97^g^
	Used low-level hyperlink categories	Yes	Yes	Yes	Yes	No^g^
**Dimension 2: navigation simplicity**							
	Constant navigational toolbar	Yes	Yes	No^g^	No^g^	Yes
	Explicit hyperlink labels	Yes	Yes	No^g^	No^g^	No^g^
	Upper left hyperlink location	Yes	Some^g^	No^g^	Some^g^	Some^g^
	Introductory content location after hyperlinks	Yes	Yes	No^g^	Yes	Yes
**Dimension 3: comprehensibility**							
	Number of words	351	586^g^	609^g^	407	551^g^
	Page length	13.7	25.0^g^	22.3^g^	15.9	24.7^g^
	Memory aids used	Yes	No^g^	No^g^	Yes	Yes
	Reading level (grade)^i^	10.9	10.2	9.4	12^g^	10.5
	Minimal inference required	Yes	No^g^	No^g^	No^g^	No^g^
	Use of dialect	Yes	No^g^	No^g^	No^g^	No^g^

^a^Each variable was split into 2 levels because there was adequate variability for each variable. One was defined as “less complex” and the other “more complex.” The demarcation for each variable was based on the relative complexity among the set of websites: the high end is “more complex” and the low end “less complex.”

^b^Schizophrenia Online Access to Resources.

^c^Schizophrenia.com.

^d^Chovil.com.

^e^National Alliance on Mental Illness.

^f^National Mental Health Association.

^g^Variable levels that were defined as more complex in this dichotomy.

^h^For reading ease, higher numbers represent better reading ease.

^i^For reading level, higher numbers represent more difficult reading level.

**Table 3:** Website performance for dichotomized^a^ Flat Explicit Design Model variables in 12 variable groupings.

19 Flat Explicit Design Model variables^a^ listed in their low-complexity form		Website complexity for each variable group^b^	N^c^	Content disclosure task: number of times ≥5 correct of 7^d^		Find contents: number of times all 3 tasks completed correctly^e^		Find contents: mean time (seconds) to correctly find contents^f^		Website ease of use rating^g^	
				n^e^(%)		n^f^(%)		Mean	Range	Mean	Median
**Dimension 1: page complexity**											
	Few navigation areas Few topic Areas Few columns	High	113	30 (26^g^)		62 (54.4^j^)		60.2^i^	10.3-207.8	3.1^h^	3
		Low	75	13 (17)		54 (72.0)		50.1	12.4-169.3	3.4	4
	Shallow depth of hierarchy Few themes presented Few display distractions Easy to moderate reading ease	High	76	12 (16^g^)		32 (42.1^j^)		65.3^j^	10.3-207.8	2.9^j^	3
		Low	112	31 (27)		84 (74.3)		50.6	12.4-169.3	3.5	4
	Fewer hyperlinks	High	150	42 (28^h^)		96 (63.6)		55.5	10.3-207.8	3.4^i^	3
		Low	38	1 (3)		20 (52.6)		58.0	21.1-169.3	2.7	3
	Used low-level hyperlink categories	High	38	6 (16)		13 (34^h^)		67.5i	22.4-150.3	2.8^i^	3
		Low	150	37 (25)		103 (68)		53.2	10.3-207.8	3.3	3.5
**Dimension 2: navigation simplicity**											
	Used a constant navigational toolbar Had moderate reading level	High	76	7 (9^j^)		39 (51.3^h^)		60.3^h^	10.3-207.8	2.9^j^	3
		Low	112	36 (32)		77 (68.1)		53.5	12.4-150.3	3.5	4
	Used explicit hyperlink labels	High	114	13 (11^j^)		52 (45.6^j^)		62.7^j^	10.3-207.8	2.8^j^	3
		Low	74	30 (40)		64 (85.3)		47.1	12.4-137.5	3.8	4
	Hyperlinks were located in upper left of the page	High	151	31 (20)		82 (52.6^j^)		59.6j	10.3-207.8	3.0^j^	3
		Low	37	12 (32)		34 (91.9)		42.8	12.4-112.1	4.0	4
	Introductory content located after hyperlinks	High	38	1 (3^i^)		20 (52.6)		58.0	21.1-169.3	2.7^i^	3
		Low	150	42 (28)		96 (63.6)		55.5	10.3-207.8	3.4	3
**Dimension 3: comprehensibility**											
	Fewer words on homepage Shorter homepage length	High	75	18 (24)		53 (70.7^h^)		51.8^g^	10.3-207.8	3.5^i^	4
		Low	113	25 (22)		63 (55.3)		58.7	15.7-169.3	3.1	3
	Memory aids were available	High	75	19 (25)		50 (65.8)		54.6	15.7-169.3	3.2	3
		Low	113	24 (21)		66 (58.4)		57.1	10.3-207.8	3.3	3
	Use of dialect Minimal inference required	High	151	31 (20)		82 (54.0^j^)		59.6^j^	10.3-207.8	3.0^j^	3
		Low	37	12 (32)		34 (91.9)		42.8	12.4-112.1	4.0	4
	Reading level ≤11th grade	High	38	6 (16)		19 (50)		62.9	10.3-207.8	3.0	3
		Low	150	37 (25)		97 (64)		54.6	12.4-169.3^g^	3.3	3

^a,b^Variables that are collinear are grouped together. Each variable was dichotomized, or split, into two levels. One was defined as “low complexity” and the other “high complexity.”

^c^The number of tasks performed on “high”- or “low”-complexity websites. N is the number of subjects times the number of websites summed across websites with low and high complexity level of the design variables, for example, if N=38 this would imply that 38 subjects viewed this level of complexity, and given there were 38 subjects total, this means that only 1 website met this criteria; if N=114 (ie, 38×3) this implies that 3 websites met this criteria.

^d^The number of times participants got >5 correct on a website. The data are separated in the table by whether the task was performed on websites with “high” or “low” complexity. Given there are 38 subjects the maximum correct is 38×5=190.

^e^The number of times participants correctly found all 3 pieces of information on a website. The data are separated in the table by whether the task was performed on websites with “high” or “low” complexity.

^f^This is based on the mean time to find information in the participants who correctly answered within the 5 minute time allotted.

^g^Significance *P*>.05

^h^Significance *P*≤.05.

^i^Significance *P*≤.01.

^j^Significance *P*≤.001.

### Assessment of the Theoretical Dimensions of the FEDM: Page Complexity, Comprehensibility, and Navigation Simplicity

The summed scores for the 3 proposed dimensions all had coefficient alphas=0.8 (page complexity alpha=0.95, Navigational Simplicity alpha=0.80, and comprehensibility alpha=0.84), indicating that the variables within each of these dimensions are internally consistent. For the usability outcomes of ability to find information, mean time to find information, and ease of website use scores, the dimensions page complexity and navigational simplicity were significantly related to more positive outcomes (*P*<.01 for all). For the fourth usability outcome of correctly identifying which information is present in a website after studying the homepage, the summary scores of Navigational Simplicity (*P*<.001) and comprehensibility (*P*=.02) were significant.

## Discussion

### Summary of Evidence

This study extends prior work with the FEDM for creating eHealth apps for individuals with SMI [23,24]. These findings indicate that the novel design variables of topic areas, navigation areas, and columns influenced the usability of websites for people with schizophrenia. The study provides support that the 19 variables in the FEDM capture important design constructs that influence usability. This evaluation provides initial support for the validity of the 3 new dimensions we created for organizing these 19 variables, that of page complexity, navigation simplicity, and comprehensibility. These dimensions focus on 3 characteristics of a design that are critical to the usability of technology, and likely the associated cognitive effort required by individuals for its use.

The written text of the tested websites was at a relatively high reading level. The calculated reading levels were potentially higher than their effective levels due to a number of words that increased the reading levels but that participants likely understood, such as “schizophrenia.” In addition, the introductory text on the homepage of SOAR was at the bottom of the page, after the hyperlinks to contents, and thus its reading level likely had less of an effect on the complexity of the page for navigation, compared with websites where the text came prior to the hyperlinks or was interspersed among the hyperlinks.

For the variable “reading ease,” low to intermediate levels were the best. Our common finding has been that, for most variables, less complexity is the best, but only within the context of certain trade-offs, as discussed later. This finding may indicate that text that is too simple may be written using more words, which makes it less efficient and more difficult to understand. Text at a slightly more advanced level may be more efficient and consequently easier for many to understand, including people with schizophrenia. Additionally, the amount of text was associated with the ability to find information, time to find information, and ease of use ratings. The lowest quantity of text was the best. Our prior research indicates that more words should be used when necessary to ensure comprehension and make meanings explicit (eg, for hyperlinks) [24]. Taken together, this group of findings is consistent with the conclusion that text efficiency is the critical variable, as long as understanding is not compromised by using wording that is too brief, at too high of a reading level, or too abstract and, consequently, not explicit.

The number of columns used to organize the information on a screen may not capture the full impact of this variable on usability. Some websites varied the number of columns used as one proceeded down a screen, whereas others were consistent throughout the screen. It is likely that information displays that change over a single screen or page require more cognitive effort to understand and navigate than those that are consistent. In prior work we found that some persons with SMI did not understand the convention that columns separate contents across rows in a table[23]. Thus, the use of columns, and other geometric conventions, can pose usability obstacles.

When designing an app, several trade-offs must be made between the dimensions of the FEDM. For example, there is a trade-off between information density and a page’s complexity. A standard approach to design is to minimize the content on a given screen, by having short, one- or two-word-long hyperlinks, relatively few hyperlinks on a screen, minimal text on navigation pages (vs final destination pages), and no more information than can fit on a single screen (ie, scrolling is not used). This approach emphasizes “paging,” going to a new page to find additional information or hyperlinks, where again the contents are minimal. This minimizes information density and a page’s layout complexity. These are 2 strengths of this design, but there are also potential shortcomings with this approach, particularly for those with SMI: (1) it requires relatively more navigation; thus, the difficulty of searching through an individual page is reduced at the cost of needing to navigate and search through more pages and a more complex hierarchy; (2) the short hyperlink labels may be enigmatic, particularly for those with SMI; and (3) the reduced information on a page may actually make it harder to understand the information being provided due to the limited context, whereas more information, to an extent, may improve users’ understanding of the contents. More information can have some advantages, but is not independent of quantity or its organization as our results indicate. Providing more information can also necessitate longer pages, which may require scrolling. Scrolling has negative consequences associated with it. Achieving this without scrolling is the ideal solution, but it may be far better to make users with SMI scroll than to navigate to a new page and through a more complex hierarchy [22].

Given the novelty of several of the complexity concepts (eg, navigation areas, topic areas), we assessed variations in ways to measure them to explore whether a particular metric might prove superior for predicting usability. All variations performed similarly using this dataset.

### Limitations

The influence on a website’s usability of users’ abilities to understand the text was potentially only grossly measured by assessing a page’s reading level and ease. It might also be that the hyperlink, versus nonhyperlink text, was far more understandable on some sites than others, and that this subcomponent of the text had a greater influence on usability.

The tasks did not assess the ability to understand content but rather the ability to find contents. This has implications for interpreting these findings. For example, pictures and diagrams may inhibit navigational efficiency; however, in the correct context, they may aid in content understanding. We examined the usability of navigational pages, not the effectiveness of content pages.

Given the number of variables to websites, it is important to acknowledge that the results might not be the same with a different set of websites. Also, the conclusions must be tempered because of the collinearity among several variables. Having said this, we have had findings consistent with these in studies that used far more websites [28].

We have conducted similar evaluations to what are presented here on websites that we designed from scratch. The work reported here was intended to evaluate these design principles on actual websites available to the public. The use of real websites means that the range of designs is more limited than if the websites were constructed solely to evaluate design principles. However, this provides evaluation of these design principles on real-world apps, which is important for understanding the significance of design to the usage of actual websites. In this context, it must be pointed out that the FEDM does not necessarily apply to apps where navigation routes are predefined by design, such as in software installation “wizards,” or similar apps where the user does not self-navigate but simply answers questions and the navigation path is preprogrammed based on responses to questions by the designer [25], and thus, in such apps, the hierarchy can be quite deep with many branching points, and still not be complex from a user’s perspective. We only tested the websites on persons who had an SMI. Consequently, we do not know the extent to which these findings are relevant to other individuals.

The participants in this study did not include younger individuals in their teens and twenties. It is possible that these findings will not be as applicable to those who are earlier in their illness, or are more familiar with technology. Preliminary analyses of our data, yet to be published, indicate that age is not as important as expertise with technology in determining which designs are more or less usable.

### Conclusions

Websites may contain vast resources; some have millions of pages [34]. Research to make such apps usable by persons with SMI, and others with cognitive impairments, who may also have limited technology experience, is still in its early stages[20,22,23,30]. A recent systematic review found only 10 studies, in addition to 3 we have conducted, which assessed barriers (and potential responses) to website use by individuals with mental illness (broadly defined) [35]. They found 42 barriers and 59 potential responses. Although some specific design responses to identified barriers do exist, and the number of evidence-based responses is growing, for many barriers, only very general guidance can be offered at present. The evidence-based guidance that has been published offers a foundation for creating more usable designs.

Given the growing use of technology-delivered health care services, a major public health challenge is to create design models for those with special cognitive needs and low technology expertise, such as persons with SMI. Our findings, coupled with the findings of others who have shown that eHealth apps designed using standard models are not usable by many with schizophrenia [22], imply that apps designed specifically to accommodate the cognitive needs of persons with SMI are needed. When this is done, these designs can be more usable, and their use is more effective and efficient than those designed using standard models. This study further highlights these conclusions. The FEDM provides a model that can be used to aid the design of eHealth apps. It has been created based on empirical findings from usability studies and developed to provide specific recommendations for creating accessible designs. The FEDM’s 3 dimensions provide practical constructs and guidance. At the simplest level, the 3 dimensions of navigational simplicity, reduced page complexity, and comprehensibility offer help in designing sites that support key tasks that users must accomplish during successful eHealth apps usage. Beyond these simple goals, the research shows the variables that contribute in each dimension allowing designers to estimate the impact of such things as the number of hyperlinks or images on a given page and how they relate to page complexity. Although some research has been conducted to identify design barriers to effective use of eHealth technologies, including our own, there is a clear need for additional research on both barriers and solutions that use rigorous experimental designs to enhance validity.

## Abbreviations

FEDM: Flat Explicit Design Model
GAS: Global Assessment Scale
SMI: severe mental illness
SOAR: Schizophrenia Online Access to Resources website
